# Mortality prediction in geriatric ICU patients with pneumonia-related sepsis: APACHE II, NEWS, and serum lactate

**DOI:** 10.17305/bb.2026.13780

**Published:** 2026-02-06

**Authors:** Ferhan Demirer Aydemir, Adil Cetin, Murat Das, Ece Unal Cetin, Ozge Kurtkulagi, Feyza Mutlay, Yavuz Beyazıt

**Affiliations:** 1 Department of Internal Medicine, Division of Intensive Care Medicine, Faculty of Medicine, Canakkale Onsekiz Mart University, Canakkale, Türkiye; 2 Department of Internal Medicine, Canakkale Mehmet Akif Ersoy State Hospital, Canakkale, Türkiye; 3 Department of Emergency Medicine, Faculty of Medicine, Canakkale Onsekiz Mart University, Canakkale, Türkiye; 4 Department of Internal Medicine, Faculty of Medicine, Canakkale Onsekiz Mart University, Canakkale, Türkiye; 5 Department of Internal Medicine, Division of Intensive Geriatrics, Faculty of Medicine, Canakkale Onsekiz Mart University, Canakkale, Türkiye; 6 Department of Internal Medicine, Division of Intensive Gastroenterology, Faculty of Medicine, Canakkale Onsekiz Mart University, Canakkale, Türkiye

**Keywords:** Sepsis, geriatric, intensive care unit, APACHE II, NEWS, lactate, mortality

## Abstract

Sepsis secondary to pneumonia is a prominent cause of intensive care unit (ICU) admissions and mortality among older adults, yet early bedside risk stratification poses significant challenges. This study aimed to evaluate the predictive value of the Acute Physiology and Chronic Health Evaluation II (APACHE II) and the National Early Warning Score (NEWS), both individually and in combination, alongside admission serum lactate levels, for predicting mortality in geriatric ICU patients with pneumonia-related sepsis. In this single-center retrospective cohort study, we analyzed patients aged 65 years and older who were admitted between January 1, 2020, and July 1, 2025. Sepsis was defined according to Sepsis-3 criteria; APACHE II (using the worst values within the first 24 hours) and NEWS (measured at ICU admission) were recorded, along with the first lactate and other biomarkers obtained within the first 24 hours. We assessed mortality predictors using logistic regression and evaluated model discrimination through receiver operating characteristic (ROC) analysis. Among the 179 patients (median age 80), the ICU mortality rate was 64.8%. Non-survivors exhibited significantly higher APACHE II and NEWS scores, as well as elevated lactate and inflammatory markers (all *P* < 0.001). In multivariable analysis, APACHE II (OR 1.130; *P* < 0.001), NEWS (OR 1.239; *P* ═ 0.003), and a history of stroke (OR 2.856; *P* ═ 0.041) were identified as independent predictors of mortality, whereas lactate did not demonstrate independent predictive capability. Although lactate improved the discrimination of a baseline clinical-laboratory model (AUC increased from 0.67 to 0.75), it offered no incremental benefit when APACHE II and NEWS were included; the combined APACHE II+NEWS model achieved the highest AUC of 0.85. Exploratory cut-offs identified very high-risk subgroups (APACHE II >21 with NEWS >8 or lactate >2 mmol/L), with mortality rates approximating 86%–87%. In conclusion, APACHE II and NEWS are robust early predictors of mortality in geriatric patients with pneumonia-related sepsis, while lactate may assist in early risk stratification but provides limited prognostic value beyond these scoring systems.

## Introduction

Community-acquired pneumonia and sepsis secondary to pneumonia are among the leading causes of intensive care unit (ICU) admissions and mortality in the geriatric population [1]. The decreased physiological reserves associated with advanced age, the higher prevalence of comorbid conditions, and a weakened immune response can complicate early recognition of clinical deterioration [2]. Therefore, accurate and timely prediction of mortality risk in geriatric patients diagnosed with sepsis secondary to pneumonia is critical for effective patient management and optimal utilization of ICU resources.

Various clinical scoring systems are commonly employed in intensive care practice to predict mortality. The Acute Physiology and Chronic Health Evaluation II (APACHE II) scoring system, which assesses both physiological deterioration and chronic disease burden, has long served as a reference for predicting ICU mortality [3]. However, its computational complexity and time-consuming nature have sparked interest in alternative scoring systems that can be applied quickly at the bedside. In this context, the National Early Warning Score (NEWS) is a well-established tool that aids in the early detection of clinical deterioration across various acute disease states [4–7]. Although the prognostic value of NEWS has been demonstrated in diverse patient populations, data on its combined use with more comprehensive ICU scoring systems in geriatric patients with pneumonia-related sepsis remain limited [8]. This study utilized the Sequential Organ Failure Assessment (SOFA) to define sepsis according to Sepsis-3 criteria, while APACHE II was employed as a prognostic scoring system to evaluate mortality risk. This choice was informed by APACHE II’s capacity to integrate acute physiological abnormalities with chronic health status, which is particularly relevant for geriatric patients with multiple comorbidities.

In addition to clinical scoring systems, biochemical markers may also play a significant role in predicting mortality associated with sepsis secondary to pneumonia in ICU patients. Serum lactate is widely used in clinical settings to assess and monitor the severity of critical illness, serving as an indirect marker of tissue hypoperfusion and metabolic stress [9–11]. Beyond its role as an inflammatory marker, lactate reflects the interplay between hemodynamic instability, microcirculatory dysfunction, and cellular metabolic alterations, which are central mechanisms in the pathophysiology of sepsis [9]. However, despite its extensive use, the prognostic performance of serum lactate may vary based on patient characteristics and clinical context, and its standalone predictive value remains contentious. This uncertainty is particularly pertinent in the geriatric population, where age-related physiological changes and a high burden of comorbidities may obscure early clinical signs of deterioration. In this context, objective biochemical parameters such as lactate may provide additional support for earlier and more accurate risk stratification.

Currently, there is limited evidence supporting the combined use of NEWS, APACHE II, and lactate as a prognostic tool for geriatric patients with pneumonia-related sepsis. Thus, integrating clinical scoring systems with objective laboratory markers may facilitate the earlier detection of high-risk subgroups and support more informed clinical judgment. This study aims to determine whether the integration of these parameters with distinct hemodynamic indicators enhances prognostication in patients with sepsis secondary to pneumonia admitted to the ICU.

## Materials and methods

This retrospective, single-center cohort study was conducted in the Medical Intensive Care Unit of Canakkale Onsekiz Mart University Hospital. We reviewed electronic medical records of geriatric patients admitted to the ICU with a diagnosis of pneumonia fulfilling sepsis secondary to pneumonia criteria between January 1, 2020, and July 1, 2025.

### Patient selection

Sepsis was defined according to Sepsis-3 criteria as sepsis secondary to pneumonia accompanied by documented organ dysfunction [12]. Inclusion criteria included age ≥65 years, a confirmed diagnosis of sepsis secondary to pneumonia in the ICU, and availability of complete blood count and biochemical parameters within the first 24 h of admission. We excluded patients who were followed for less than 24 h, those with incomplete clinical or laboratory data, those receiving immunosuppressive therapy, and those with active hematologic malignancies. According to Sepsis-3 criteria, sepsis was operationalized as suspected or confirmed infection accompanied by organ dysfunction, indicated by an increase in the SOFA score of ≥2 points. We assumed a baseline SOFA score of zero in patients without documented pre-existing organ dysfunction, consistent with the original Sepsis-3 definition. SOFA scores were calculated using the worst physiological and laboratory values within the first 24 h after ICU admission.

Pneumonia was defined based on a combination of clinical, radiological, and microbiological criteria, including new or progressive pulmonary infiltrates on chest imaging along with at least one clinical sign of infection (fever, leukocytosis/leukopenia, purulent respiratory secretions, or worsening respiratory status). Patients with both community-acquired and hospital-acquired pneumonia were included, while COVID-19-related pneumonia was excluded to avoid potential confounding related to distinct pathophysiological and clinical characteristics.

**Figure 1.**
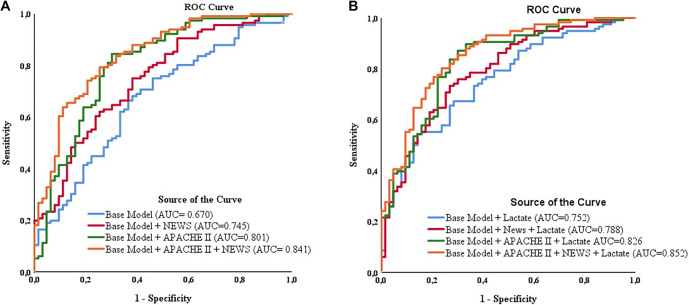
**ROC curves comparing discrimination of prognostic models for ICU mortality.** (A) Models excluding lactate (base model; base model + NEWS; base model + APACHE II; base model + NEWS + APACHE II). (B) The same nested models after addition of lactate. The base model comprised age, sex, procalcitonin, CRP, SII, and albumin. AUCs are displayed on the plot; statistical comparisons between AUCs are reported in Table 4. Abbreviations: ROC: Receiver operating characteristic; ICU: Intensive care unit; NEWS: National Early Warning Score; APACHE II: Acute Physiology and Chronic Health Evaluation II; CRP: C-reactive protein; SII: Systemic immune–inflammation index; AUCs: Areas under the ROC curve.

### Data collection and variables

We retrieved demographic information, clinical severity scores, and laboratory measurements from the hospital information system and ICU documentation. The Systemic Immune-Inflammation Index (SII) was calculated using platelet, neutrophil, and lymphocyte counts obtained within the first 24 h after diagnosis. APACHE II and NEWS scores, along with laboratory parameters including lactate levels, were recorded at ICU admission and within the first 24 h. These variables were considered baseline predictors of mortality. APACHE II scores were calculated using the worst physiological and laboratory values obtained within the first 24 h after ICU admission, following the original APACHE II methodology. NEWS scores were calculated based on vital signs recorded at ICU admission. Mechanical ventilation and vasopressor use were evaluated separately as indicators of disease severity and clinical course during the ICU stay, rather than as baseline predictors. Serum lactate levels were measured from arterial blood gas samples, with the first lactate value obtained within the first 24 h after ICU admission used for analysis. Patients were classified into discharge and mortality groups based on ICU outcomes.

Sample size estimation was performed using G*Power software (version 3.1.9.7). The assumed effect size was derived from previous studies on the prognostic value of lactate and severity scores in geriatric ICU patients, as reported by Duzgun and Kalin [13]. The calculation assumed a moderate effect size and an event proportion comparable to the mortality rate reported in the reference cohort. Using a logistic regression model with an α error probability of 0.05 and a statistical power (1-β) of 0.80, the minimum required sample size was calculated at 160 patients. The final study population (*n* ═ 179) exceeded this threshold, indicating adequate statistical power for the primary analyses. During the study period, 247 geriatric patients admitted to the ICU with suspected pneumonia-related sepsis were screened. After excluding patients with incomplete clinical or laboratory data (*n* ═ 38), ICU stays of less than 24 h (*n* ═ 19), immunosuppressive therapy or active hematologic malignancy (*n* ═ 11), a total of 179 patients were included in the final analysis.

### Ethical statement

The study was conducted in accordance with the Declaration of Helsinki and approved by the university’s Institutional Review Board. Due to the retrospective nature of the study, informed consent was waived, and all patient data were anonymized (approval date: 24.07.2025; decision no: GOKAEK/2025-260).

### Statistical analysis

All statistical analyses were performed using the Statistical Package for the Social Sciences (SPSS) 19.0 for Windows (IBM Corp., Armonk, NY, USA) and R software version 3.6.2. A *P* value of less than 0.05 was deemed statistically significant. The distribution of continuous variables was evaluated using the Shapiro–Wilk test. Normally distributed variables were reported as mean ± standard deviation (SD), whereas non-normally distributed variables were presented as median (interquartile range, IQR). Between-group comparisons were conducted using either the independent samples *t*-test or the Mann–Whitney *U* test, as appropriate. Categorical variables were assessed using the chi-square test or Fisher’s exact test. Logistic regression analyses were performed to identify factors associated with mortality. Variables with *P* < 0.01 from the univariable analysis, along with clinically relevant covariates—such as age, sex, the Confusion, Urea, Respiratory rate, Blood pressure, and age ≥65 years score (CURB-65), pH, and history of stroke—were incorporated into the multivariable logistic regression model. To prevent the inclusion of post-admission, time-dependent treatments in baseline prognostication, organ support variables (mechanical ventilation and vasopressor use) were excluded from the regression models. Multicollinearity among predictors was evaluated using tolerance and variance inflation factors (VIF), revealing no significant issues (minimum tolerance = 0.916; maximum VIF = 1.092). Model calibration was assessed using the Hosmer–Lemeshow test and a logistic calibration regression of mortality on the logit of predicted probabilities, yielding calibration intercept and slope, and further examined with a decile-based calibration assessment. Internal validation was conducted through bootstrap resampling (1,000 iterations) to estimate optimism and obtain optimism-corrected area under the curve (AUC) and calibration slope. Continuous forms of APACHE II, NEWS, and lactate served as primary predictors in multivariable models to preserve information and maintain statistical power. Dichotomized thresholds were applied solely for secondary and exploratory analyses to enhance clinical interpretability, based on previously reported or clinically meaningful cut-off values. Given the multiple comparisons, analyses beyond the primary multivariable models (including ROC-derived cut-offs, risk stratification, and univariable screening) were considered exploratory; thus, *P* values were interpreted cautiously without multiplicity adjustment. The functional forms of continuous predictors were assessed prior to model inclusion. Prognostic performance was evaluated using ROC curves and AUC comparisons (Figure 1). Optimal cut-off values for APACHE II and NEWS were identified through ROC analysis by maximizing the Youden index (sensitivity + specificity -- 1). The DeLong test was subsequently employed for pairwise comparison of area under the receiver operating characteristic curves (AUROCs), with a *P* value <0.05 considered statistically significant.

Missing data were addressed using a complete-case analysis approach. Patients with missing critical clinical or laboratory variables necessary for regression and ROC analyses were excluded prior to model construction. Among the 247 screened patients, 38 (15.4%) were excluded due to incomplete clinical or laboratory data. The missingness pattern was examined and primarily related to unavailable laboratory measurements rather than outcome status. Although complete-case analysis may introduce potential selection bias, the proportion of excluded cases was minimal, and the baseline characteristics of included patients were deemed representative of the target population.

## Results

### Patient characteristics and clinical severity

A total of 179 patients were included in the study. The median age was 80 years, with no significant differences in age or sex distribution between groups (*P* ═ 0.793 and *P* ═ 0.382). Table 1 presents baseline demographic data and clinically relevant comorbidities associated with disease severity and mortality risk in sepsis (Table 1). Survivors (*n* ═ 63) exhibited significantly lower disease severity, with APACHE II and NEWS scores of 19.0 and 7.0, respectively, compared to 30.5 and 10.0 in the mortality group (both *P* < 0.001). Comorbidities were prevalent overall; however, only a history of stroke was significantly associated with mortality (25.9% vs. 12.7%; *P* ═ 0.029).

**Table 1:** Demographic, clinical, laboratory, and ICU-course characteristics of the study population by survival status

**Variable**	**All patients (*n* ═ 179)**	**Survivors (*n* ═ 63)**	**Non-survivors (*n* ═ 116)**	***P* value**
Age, years, median (IQR)	80.0 (73.0–87.0)	80.0 (73.5–85.5)	80.0 (73.0–88.0)	0.793
Sex, male/female, *n* (%)	96 (53.6) / 83 (46.4)	31 (49.2) / 32 (50.8)	65 (56.0) / 51 (44.0)	0.382
APACHE II	28.0 (20.0–35.5)	19.0 (13.0–27.0)	30.5 (25.5–37.0)	<0.001
NEWS	9.0 (7.0–11.0)	7.0 (6.0–10.0)	10.0 (8.0–12.0)	<0.001
CURB-65	3.0 (3.0–4.0)	3.0 (2.0–4.0)	3.0 (3.0–4.0)	0.015
PSI	154.0 (125.5–186.0)	158.0 (128.5–212.5)	152.5 (121.0–183.5)	0.325
SII (×10^3^)	2953.9 (1435.0–6420.8)	2747.0 (1380.2–6213.2)	2997.5 (1483.9–6515.4)	0.614
Comorbidities				
Diabetes mellitus, *n* (%)	47 (26.3)	18 (28.6)	29 (25.0)	0.604
Coronary artery disease, *n* (%)	34 (19.0)	14 (22.2)	20 (17.2)	0.417
Stroke, *n* (%)	38 (21.2)	8 (12.7)	30 (25.9)	0.029
WBC (×10^9^/L)	12.8 (9.2–17.7)	11.0 (8.9–15.6)	14.7 (9.6–18.6)	0.013
Neutrophil (×10^9^/L)	11.1 (7.4–16.0)	8.8 (7.2–14.3)	12.8 (8.1–17.0)	0.029
Procalcitonin (ng/mL)	1.9 (0.6–8.8)	0.9 (0.3–2.4)	3.3 (1.1–12.0)	<0.001
Ferritin (ng/mL)	280.0 (120.0–704.5)	226.0 (86.5–458.5)	304.5 (151.0–801.0)	0.015
Base excess (mmol/L)	--2.2 (--7.3 to 2.3)	--0.3 (--4.9 to 4.1)	--2.9 (--7.7 to 1.45)	0.014
Albumin (g/dL)	2.9 (2.5--3.3)	3.1 (2.8--3.3)	2.8 (2.4--3.1)	<0.001
C-Reactive Protein (mg/L)	147.0 (82.5--234.0)	122.0 (64.0--230.0)	159.0 (93.4--236.5)	0.133
Lactate (mmol/L)	1.8 (1.3–2.9)	1.4 (1.1–1.9)	2.3 (1.4–3.5)	<0.001
Mechanical ventilation, *n* (%)	116 (64.8)	14 (22.2)	102 (87.9)	<0.001
Inotrope/vasopressor use, *n* (%)	77 (43.0)	10 (15.9)	67 (57.8)	<0.001
Length of ICU stay, days	9.0 (5.0–16.0)	8.0 (5.0–10.5)	9.0 (4.5–20.5)	0.130

Abbreviations: APACHE II: Acute Physiology and Chronic Health Evaluation II; NEWS: National Early Warning Score; CURB-65: Confusion, Urea, Respiratory rate, Blood pressure, age ≥65; PSI: Pneumonia Severity Index; SII: Systemic Immune-Inflammation Index; WBC: White blood cell count; ICU: Intensive care unit; IQR: Interquartile range.

### Laboratory findings

Markers of systemic inflammation and metabolic stress were consistently elevated in the mortality group. White blood cell count (WBC) was 14.7 vs. 11.0 (*P* ═ 0.013), neutrophil count was 12.8 vs. 8.8 (*P* ═ 0.029), procalcitonin levels were 3.3 vs. 0.9 ng/mL (*P* < 0.001), and ferritin levels were 304.5 vs. 226.0 (*P* ═ 0.015), all significantly associated with mortality. Additionally, reduced base excess (–2.9 vs. –0.3; *P* ═ 0.014) and elevated lactate levels (2.3 vs. 1.4 mmol/L; *P* < 0.001) indicated tissue hypoperfusion in the mortality group. Other hematologic and biochemical parameters did not show significant differences.

### ICU support requirements

The need for organ support reflected the clinical trajectory and severity of illness during the ICU stay and was strongly correlated with mortality. Mechanical ventilation was necessary for 87.9% of non-survivors compared to 22.2% of survivors (*P* < 0.001). Similarly, vasopressor use was more frequent in the mortality group (57.8% vs. 15.9%; *P* < 0.001) (Table 1).

### Univariate and multivariate analyses

The primary analyses utilized continuous values of APACHE II, NEWS, and lactate, while dichotomized cut-offs were presented as supplementary analyses for clinical interpretability. In univariate analysis, APACHE II, NEWS, and lactate were the most strongly associated variables with mortality (all *P* ≤ 0.001), with stroke history emerging as an additional risk factor (OR=2.398; *P* ═ 0.044). In multivariate analysis, APACHE II (OR=1.130; *P* < 0.001), NEWS (OR=1.239; *P* ═ 0.003), and stroke history (OR=2.856; *P* ═ 0.041) remained independently associated with mortality, whereas lactate lost statistical significance (*P* ═ 0.108) (Table 2). Calibration analysis demonstrated excellent agreement between predicted and observed risks, with a calibration intercept of 0.000 and a slope of 1.000. The model also exhibited acceptable goodness-of-fit (Hosmer–Lemeshow χ^2^ ═ 6.082, degrees of freedom (df) ═ 8, *P* ═ 0.638).

**Table 2:** Univariate and multivariate logistic regression analysis of ICU mortality

**Variable**	**Univariate OR (95% CI)**	***P* value**	**Multivariate OR (95% CI)**	***P* value**
Age	1.004 (0.968–1.041)	0.837	1.019 (0.974--1.066)	0.412
Sex [male vs female (reference)]	0.760 (0.411–1.406)	0.382	0.541 (0.246--1.188)	0.126
APACHE II	1.124 (1.080–1.170)	<0.001	1.130 (1.075–1.178)	<0.001
NEWS	1.269 (1.134–1.419)	<0.001	1.239 (1.073–1.430)	0.003
CURB-65	1.493 (1.058–2.107)	0.022	1.208 (0.791--1.845)	0.382
PSI	0.996 (0.989–1.002)	0.212		
SII	1.000 (1.000–1.000)	0.673		
pH	0.081 (0.005–1.355)	0.081	0.800 (0.015--25.963)	0.800
PaO2	1.007 (0.996–1.018)	0.216		
PaCO2	0.993 (0.974–1.013)	0.493		
Lactate	1.665 (1.239–2.237)	0.001	1.254 (0.922–1.706)	0.149
Stroke	2.398 (1.025–5.612)	0.041	2.856 (1.044--7.813)	0.041

Variables with a *P* value less than 0.01 in the univariable analysis, along with prespecified clinically relevant covariates, were included in the multivariable model. ORs are reported with 95% CIs. Statistical significance was established at a threshold of *P* < 0.05. Abbreviations: APACHE II: Acute Physiology and Chronic Health Evaluation II; NEWS: National Early Warning Score; CURB-65: Confusion, Urea, Respiratory rate, Blood pressure, age ≥65; PSI: Pneumonia Severity Index; SII: Systemic Immune-Inflammation Index; ORs: Odds ratios; CIs: Confidence intervals.

### Combined APACHE and NEWS stratification

Among patients with APACHE scores ≤21 and NEWS scores ≤8, the mortality rate was 17.4% (Table 3). This rate increased to 37.9% when NEWS scores exceeded 8 within the same APACHE category, although this difference lacked statistical significance (*P* ═ 0.112). In contrast, patients with APACHE scores >21 and NEWS scores ≤8 faced a significantly elevated mortality risk (*P* ═ 0.001), while those with both APACHE >21 and NEWS >8 constituted the highest-risk subgroup, exhibiting a mortality rate of 85.6% (*P* < 0.001). Adjusted analyses corroborated this hierarchical risk pattern.

**Table 3:** Combined APACHE II–NEWS and APACHE II–lactate risk stratification for mortality

**Group**	**Deaths / Total n (%)**	**Crude OR (95% CI)**	***P* value**	**Adjusted OR (95% CI)**	***P* value**
APACHE II ≤21 and NEWS ≤8	4/23 (17.4)	Reference	-	Reference	-
APACHE II ≤21 and NEWS >8	11/29 (37.9)	2.903 (0.781–10.796)	0.112	2.783 (0.738–10.490)	0.131
APACHE II >21 and NEWS ≤8	24/37 (64.9)	8.769 (2.458–31.290)	0.001	9.211 (2.539–33.412)	0.001
APACHE II >21 and NEWS >8	77/90 (85.6)	28.135 (8.240–96.061)	<0.001	30.970 (8.853–108.341)	<0.001
APACHE II ≤21 and Lactate ≤2 mmol/L	8/39 (20.5)	Reference	-	Reference	-
APACHE II ≤21 and Lactate >2 mmol/L	7/13 (53.8)	4.521 (1.185–17.249)	0.027	4.135 (1.070–15.970)	0.040
APACHE II >21 and Lactate ≤2 mmol/L	47/65 (72.3)	10.118 (3.920–26.118)	<0.001	10.868 (4.134–28.568)	<0.001
APACHE II >21 and Lactate >2 mmol/L	54/62 (87.1)	26.156 (8.928–76.630)	<0.001	27.046 (9.135–80.075)	<0.001

Data are presented as the number of deaths per total number of patients (%). Crude and adjusted ORs with 95% CIs were calculated using logistic regression analysis. The adjusted models accounted for age, sex, and clinically relevant covariates. Abbreviations: APACHE II: Acute Physiology and Chronic Health Evaluation II; NEWS: National Early Warning Score; CI: Confidence intervals; ORs: Odds ratios.

### Combined APACHE and lactate stratification

In patients with APACHE scores ≤21 and lactate levels ≤2, the mortality rate was 20.5% (Table 3). Conversely, lactate levels exceeding 2 significantly raised mortality to 53.8% (*P* ═ 0.027). For patients with APACHE scores >21, mortality escalated to 72.3% (*P* < 0.001), with the APACHE >21 and lactate >2 subgroup representing the highest-risk population at 87.1% (*P* < 0.001). This trend persisted in multivariable models. These risk-stratified cutoff analyses were performed as secondary, exploratory analyses to enhance clinical interpretability.

### Model performance analysis

The baseline prognostic model exhibited modest performance (AUC=0.67). The inclusion of lactate significantly improved discrimination (AUC=0.75; *P* ═ 0.011) (Table 4). The incorporation of NEWS further enhanced performance (AUC=0.79), with this improvement remaining significant when combined with lactate (*P* ═ 0.035). Although adding APACHE increased the AUC, the incremental contribution of lactate was not statistically significant (*P* ═ 0.128). The final model, which included both NEWS and APACHE, achieved the highest AUC (0.85), but lactate no longer provided additional benefit (*P* ═ 0.369). Bootstrap internal validation (1,000 resamples) indicated that the base model (age, sex, procalcitonin, CRP, SII, and albumin) demonstrated poor discrimination after optimism correction (AUC 0.503; apparent 0.571) with substantial overfitting (calibration slope 0.393). Adding NEWS improved performance (optimism-corrected AUC 0.653; apparent 0.696; slope 0.766), and the addition of APACHE II yielded good discrimination with minimal optimism (optimism-corrected AUC 0.765; apparent 0.795; slope 0.847). The combined model (base + NEWS + APACHE II) maintained good discrimination after optimism correction (AUC 0.804; apparent 0.836) with a calibration slope of 0.828, indicating mild overfitting.

**Table 4:** Comparative discriminative performance of prognostic models with and without lactate

**Prognostic model**	**AUC without lactate (95% CI)**	**AUC with lactate (95% CI)**	**ΔAUC**	**SE Dif.**	**Lower 95% CI**	**Upper 95% CI**	**Z statistic**	***P* value**
Base model*	0.670 (0.588–0.753)	0.752 (0.680–0.825)	--0.082	0.032	--0.145	--0.018	--2.532	0.011
Base model + NEWS	0.745 (0.670–0.820)	0.788 (0.719–0.858)	--0.043	0.020	--0.083	--0.003	--2.106	0.035
Base model + APACHE II	0.801 (0.730–0.873)	0.826 (0.762–0.891)	--0.025	0.016	--0.057	0.007	--1.522	0.128
Base model + NEWS + APACHE II	0.841 (0.780–0.901)	0.852 (0.794–0.911)	--0.011	0.012	--0.036	0.013	--0.898	0.369

Values are expressed as AUC with 95% CIs. Pairwise comparisons of AUCs were conducted using the DeLong method. ΔAUC was determined by subtracting the AUC of the model without lactate from the AUC of the model with lactate; thus, negative ΔAUC values indicate enhanced discrimination following the inclusion of lactate. The base model comprised age, sex, procalcitonin, CRP, SII, and albumin. Abbreviations: NEWS: National Early Warning Score; APACHE II: Acute Physiology and Chronic Health Evaluation II; SE Dif: Standard error of the difference; CI: Confidence intervals; CRP: C-reactive protein; SII: Systemic Immune-Inflammation Index; ΔAUC: Change in area under the curve; AUC: Area under the curve.

## Discussion

Sepsis secondary to pneumonia represents a significant global healthcare challenge, associated with heightened morbidity and mortality rates [14]. This condition involves complex pathophysiological processes and necessitates aggressive early intervention, particularly in the elderly population [15]. This study assessed the combined prognostic value of clinical and biochemical parameters, alongside indicators of physiological derangement, in predicting mortality among geriatric patients with pneumonia-related sepsis admitted to the ICU of a tertiary care hospital. Our findings revealed that both APACHE II and NEWS scores were strongly correlated with mortality, as well as the necessity for mechanical ventilation and inotropic support, ranking among the most potent clinical predictors of death. Additionally, serum lactate levels significantly enhanced risk stratification, especially when interpreted alongside clinical scoring systems. These results suggest that early and accurate risk stratification in geriatric patients with pneumonia-related sepsis can substantially inform clinical decision-making and facilitate timely, targeted patient management.

In geriatric patients, the response to infections may be attenuated and delayed due to diminished physiological reserves, increased prevalence of comorbidities, and an impaired immune response [2, 16]. This combination complicates early detection of clinical deterioration, thereby elevating morbidity and mortality risks. Although several risk stratification tools such as the Pneumonia Severity Index (PSI), Frailty Index (FI), and CURB-65 are commonly employed for this purpose, these scoring systems may exhibit limitations when applied to elderly patients due to factors such as comorbidities, age-related physiological changes, and practical implementation challenges [17–20]. Consequently, there is an increasing demand for objective and validated prognostic tools that can be utilized at the time of hospitalization in geriatric patients with pneumonia-related sepsis. The primary aim of this study was to investigate whether widely recognized clinical scoring systems, like APACHE II and NEWS, combined with a readily accessible biochemical marker, such as serum lactate, could enhance risk stratification in this patient population.

Our study demonstrated that both APACHE II and NEWS scores were significantly higher in the mortality group. APACHE II, developed by Knaus et al., is a comprehensive severity-of-illness scoring system widely utilized to quantify acute physiological derangement and chronic health status in critically ill patients [21]. It assesses both physiological parameters and chronic health status [22]. In our analysis, the persistence of APACHE II as an independent predictor of mortality in both univariate and multivariate logistic regression analyses underscores its capacity to reflect disease severity in geriatric patients with pneumonia-related sepsis. The use of an APACHE II threshold >21, in particular, facilitates the clear differentiation of high-risk patient subgroups, enhancing the clinical utility of this score. However, while APACHE II effectively conveys overall illness severity, its performance may vary when employed as a standalone mortality prediction tool in a heterogeneous elderly population. This limitation is particularly pronounced in clinical environments characterized by significant variability in comorbidity burden and disease presentation. Nonetheless, in terms of mortality prediction, the APACHE II score is neither highly sensitive nor specific. A key disadvantage of this scoring system is that many patients present with multiple comorbidities, complicating their categorization under a single primary diagnostic category [23]. In this context, integrating the NEWS score with APACHE II appears to be a rational and complementary approach. Unlike APACHE II, NEWS can be readily applied upon hospitalization, is based on bedside vital signs, and aims to identify clinical deterioration at an early stage. This capability is especially crucial in elderly patients with pneumonia-related sepsis, where early physiological deterioration may be subtle and easily overlooked. Thus, the combined use of APACHE II and NEWS offers a more comprehensive assessment of both baseline disease severity and evolving clinical instability. While APACHE II, NEWS, and lactate were evaluated at ICU admission and represent baseline prognostic factors, mechanical ventilation and vasopressor requirements were interpreted as dynamic markers of disease progression rather than primary admission predictors.

Early warning systems (EWS) have been utilized in medical practice for decades to facilitate the early recognition of clinical deterioration [24, 25]. Among the various EWS, the NEWS was proposed and validated by the Royal College of Physicians in 2012, demonstrating superior performance compared to other systems in identifying patients at risk for ICU admission and mortality [26]. Our study identified a strong association between the NEWS score and mortality, with it remaining an independent predictor in multivariate analysis. This indicates that NEWS is an effective risk assessment tool for geriatric patients with sepsis secondary to pneumonia. A significant finding of our research is that NEWS, particularly when combined with the APACHE II score, enhances clinical decision-making at the point of admission. Specifically, patients with both APACHE II scores greater than 21 and NEWS scores exceeding 8 represent a subgroup with the highest mortality rates, suggesting that these two scores are complementary, as they reflect different aspects of clinical severity. While APACHE II is more indicative of physiological deterioration and chronic disease burden, NEWS captures acute clinical instability.

Although both APACHE II and NEWS have been extensively evaluated as prognostic markers for mortality and adverse ICU outcomes, most prior studies have assessed these risk stratification systems independently rather than in a combined framework. Wang et al. [27] recently demonstrated that modified NEWS and APACHE II exhibited higher discriminative power in predicting hospital and 90-day mortality among ICU patients. Similarly, Yu et al. [28] found that elevated APACHE II and NEWS scores could effectively assess the risk of death in elderly emergency patients with critical illnesses.

Lactate levels are frequently employed in the prognostic evaluation of critical illnesses, serving as an indirect marker of tissue hypoperfusion and cellular metabolic stress [29]. In our study, lactate levels were significantly elevated in the deceased group and showed a strong association with mortality in univariate analysis. However, when lactate was evaluated alongside APACHE II, NEWS, and other clinical severity indices in multivariate analysis, it did not remain an independent predictor of mortality. This suggests that lactate should not be viewed as an isolated prognostic indicator but rather as a marker to be interpreted within a clinical context. Nevertheless, in ROC analyses, the inclusion of lactate substantially increased the discriminatory power of the baseline model. The observed increase in AUC values with the addition of lactate to the baseline model—including age, sex, and inflammatory markers—indicates that lactate significantly contributes to early risk stratification. However, the lack of additional contribution from lactate in final models that included both NEWS and APACHE II suggests that these clinical scores effectively encapsulate advanced disease severity. This finding implies that lactate may be particularly useful in early stages when clinical scores are either limited or have not fully escalated.

The combination of APACHE II and lactate also yielded notable results in our study. Patients with APACHE II scores of 21 or lower and lactate levels exceeding 2 mmol/L were associated with increased mortality, while those with APACHE II scores above 21 and elevated lactate levels exhibited even higher mortality rates. This finding indicates that lactate may provide additional prognostic information, especially in patients at intermediate risk. Consistent with our findings, Duzgun et al. [13] showed that lactate levels at admission were independently associated with short-term mortality in geriatric ICU patients, providing significant early risk stratification, although its prognostic performance did not surpass that of established severity scores such as APACHE II and SOFA. Similarly, Cao et al. [30] demonstrated that the combination of lactate levels, lactate clearance, and APACHE II score more accurately predicts short-term outcomes in critically ill patients.

While this study is one of the first to investigate the combined prognostic value of NEWS, APACHE II, and lactate in geriatric sepsis secondary to pneumonia, several limitations warrant consideration. First, the relatively limited number of patients included may have reduced statistical power. Second, the retrospective design introduces inherent biases, limiting the ability to establish causal relationships. Third, as a single-center study, the generalizability of our findings to broader and more diverse patient populations may be restricted. Fourth, lactate levels were measured only at ICU admission; thus, serial lactate measurements or lactate clearance over time were not assessed, which may have hindered the evaluation of dynamic changes in tissue perfusion and their potential prognostic impact. Fifth, the combined analyses of APACHE–NEWS and APACHE–lactate risk stratification resulted in sparse cells in some strata, yielding unstable odds ratio estimates with wide 95% confidence intervals; therefore, these subgroup findings should be considered exploratory and warrant confirmation in larger cohorts. Additionally, other established risk stratification tools, such as SOFA, were not included in the analysis, which may have offered further insights into the prognostic performance of combined scoring approaches.

## Conclusion

In conclusion, our study demonstrates that clinical and biochemical risk assessment tools, in conjunction with markers of physiological derangements, are essential for predicting mortality associated with sepsis secondary to pneumonia in geriatric ICU patients. The integrated evaluation of APACHE II and NEWS scores facilitates timely detection of high-risk patient groups. Lactate levels, particularly when used alongside these clinical scores, emerge as a complementary biomarker that enhances risk stratification. These findings suggest that adopting a multidimensional and integrated prognostic approach at the time of ICU admission may improve clinical decision-making processes and patient management in sepsis secondary to pneumonia.

## Data Availability

Data are available from the corresponding author upon reasonable request.
